# Can impressive ‘pathological’ ST-T changes be a normal variant?

**DOI:** 10.1093/ehjcr/ytad611

**Published:** 2023-12-01

**Authors:** Ivana Sopek Merkaš, Nenad Lakušić

**Affiliations:** Department of Cardiology, Special Hospital for Medical Rehabilitation Krapinske Toplice, Gajeva 2, Krapinske Toplice 49217, Croatia; Department of Cardiology, Special Hospital for Medical Rehabilitation Krapinske Toplice, Gajeva 2, Krapinske Toplice 49217, Croatia; Department of Clinical Medicine, Faculty of Dental Medicine and Health Osijek, J. J. Strossmayer University of Osijek, Osijek 31000, Croatia; Department of Internal Medicine, Family Medicine and History of Medicine, Faculty of Medicine Osijek, J. J. Strossmayer University of Osijek, Osijek 31000, Croatia

## Case

**Figure 1:**
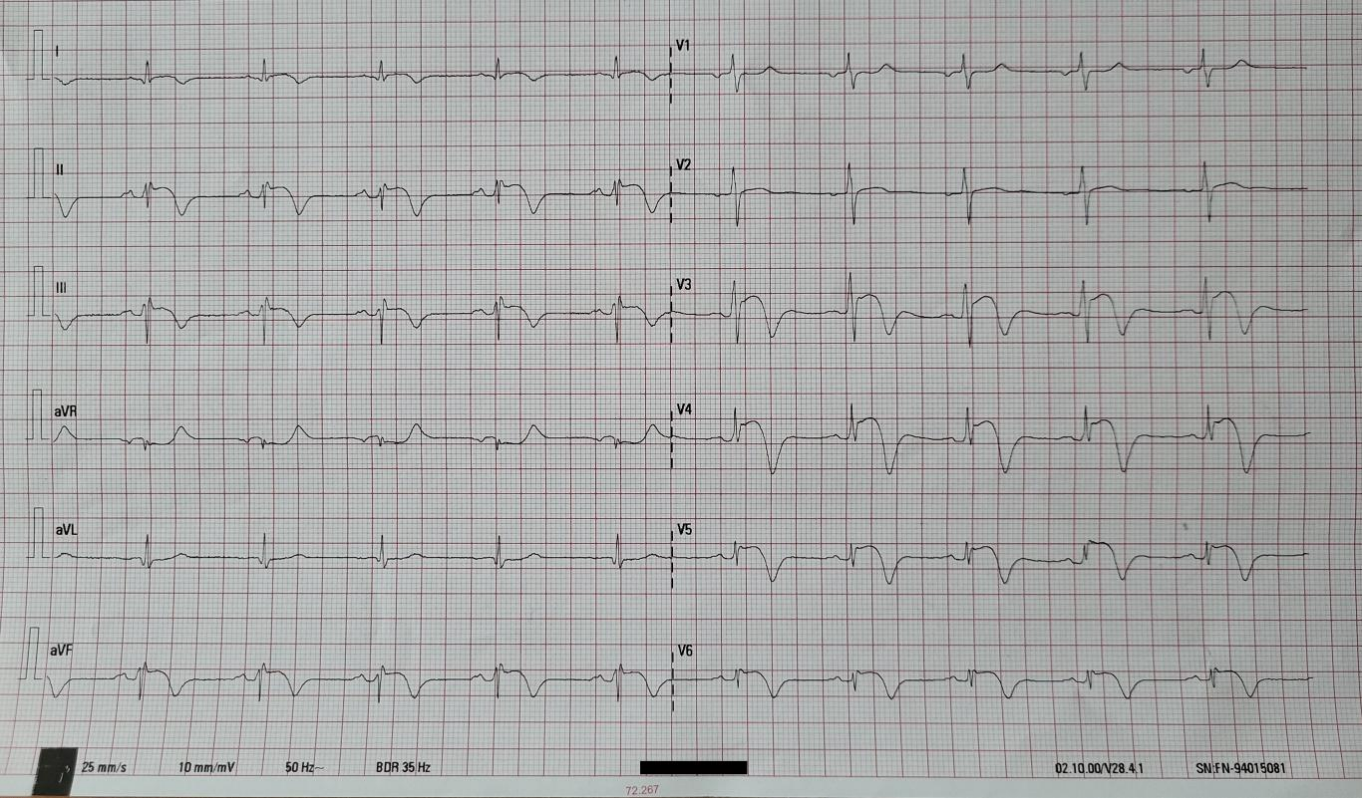
Pathological ECG changes.

A 58-year-old healthy male, with no prior medical history, underwent a routine preventive medical examination. A 12-lead electrocardiogram (ECG) was obtained and is shown in the *Figure 1*. He is an active recreational athlete, engaging in table tennis approximately three times a week for 90 min each session. He reported no symptoms or limitations in his daily activities before and at the time of the examination. The patient has a moderate risk of cardiovascular disease (CVD), with a 3–4% SCORE2 risk on the chart for countries at high CVD risk. His body mass index is within the normal range at 22 kg/m², he is a non-smoker, and he has a negative family medical history regarding CVD.

## Question 1 What diagnosis is *most closely* represented in this ECG?

- Pericarditis
- Stress-induced cardiomyopathy
- Myocardial infarction with ST-elevation (STEMI)
- Arrhythmogenic right ventricular dysplasia (ARVD)
- Hypertrophic cardiomyopathy (HCM)

The correct answer is C.

## Discussion and explanation

While all the provided answers are being considered in the differential diagnosis, it is noteworthy that the patient has no documented instances of sudden cardiac death in the family, has not experienced recent stressful events, and has been free from infections. Therefore, the provisional working diagnosis leans STEMI involving the anterior and inferolateral walls (potentially associated with the left main coronary artery). Nevertheless, the absence of any patient symptoms, the sustained amplitude of R waves in all leads where ST-T changes are recorded, and the absence of ST-elevation in aVR collectively challenge the diagnosis of STEMI.

## Question 2 What are the optimal urgent diagnostic procedures for this patient?

- Urgent coronary angiography
- Measurement of hs-troponin level and serial recording of ECG
- Echocardiography exam
- B + C
- No urgent diagnostic procedures are required—the patient has no symptoms

The correct answer is D.

## Discussion and explanation

Despite the absence of symptoms, it was rational to measure the concentration of high-sensitivity troponin (hs-troponin), accompanied by consecutive ECG recordings. The hs-troponin levels were within the normal range in the two consecutive samples, and the ECG showed no changes. Echocardiography showed a structurally and functionally normal heart. Both ventricles were non-dilated, without hypertrophy or segmental contractility disorders. The ejection fraction was normal, valvular disease was ruled out, and there was no evidence of pericardial effusion. Given that the patient did not meet most of the criteria for acute coronary syndrome, there was no indication for an urgent coronary angiography.

## Question 3 After exclusion of acute coronary syndrome, what is the rational further diagnostic algorithm?

- Exercise stress test
- Coronary angiography
- Multi-slice computed tomography-coronary angiography (MSCT-CA)
- Cardiac magnetic resonance imaging (MRI)
- C + D

The correct answer is E.

## Discussion and explanation

With a less invasive MSCT-CA, obstructive coronary disease was excluded, while cardiac MRI ruled out pathomorphological changes in the myocardium of ventricles and other cardiac structures. Over a six-month follow-up period, the patient’s ECG remained unchanged, and he remained asymptomatic.

Abnormalities in the ST-segment and T wave are common, often reflect issues with ventricular repolarization and frequently associated with various well-defined medical conditions.^1^ Despite a detailed literature search,^2^ no described pathological conditions were found to explain the observed ECG changes. Consequently, we conclude this case presentation with the rhetorical question: ‘Can impressive “pathological” ST-T changes be a normal variant?’

## Data Availability

The data underlying this article are available in the article and in its online supplementary material.

## Notes

**Consent:** Direct written consent was obtained by the patient for this manuscript.

**Funding:** No funding was involved in the development of this manuscript.
